# Supplementary material for: Top-down identification of keystone taxa in the microbiome
Source: Nat Commun. 2023 Jul 4;14:3951. doi: 10.1038/s41467-023-39459-5 (PMC10319726; doi:10.1038/s41467-023-39459-5)
Supplement: Supplementary file 1 — Supplementary Information [file 41467_2023_39459_MOESM1_ESM.pdf]

Supplementary Information for:
Top-down identification of keystone taxa in the microbiome

Guy Amit and Amir Bashan

Contents

Supplementary Figures and Table . . . 2
Supplementary Figure 1 . . . 2
Supplementary Figure 2 . . . 2
Supplementary Figure 3 . . . 3
Supplementary Figure 4 . . . 3
Supplementary Figure 5 . . . 3
Supplementary Figure 6 . . . 3
Supplementary Figure 7 . . . 3
Supplementary Figure 8 . . . 3
Supplementary Figure 9 . . . 3
Supplementary Figure 10 . . . 3
Supplementary Figure 11 . . . 3
Supplementary Figure 12 . . . 3
Supplementary Figure 13 . . . 3
Supplementary Figure 14 . . . 3
Supplementary Figure 15 . . . 3
Supplementary Figure 16 . . . 3
Supplementary Figure 17 . . . 3
Supplementary Figure 18 . . . 3
Supplementary Figure 19 . . . 3
Supplementary Figure 20 . . . 3
Supplementary Figure 21 . . . 3
Supplementary Figure 22 . . . 3
Supplementary Figure 23 . . . 3
Supplementary Figure 24 . . . 3
Supplementary Figure 25 . . . 3
Supplementary Figure 26 . . . 3
Supplementary Table 1 . . . 25
Supplementary Software . . . 26
Directed Barabási-Albert networks . . . 26
Empirical presence-abundance interrelation measures, D_1, D_2, Q . . . 27
Longitudinal EPI, L . . . 30

Supplementary Figures and Table

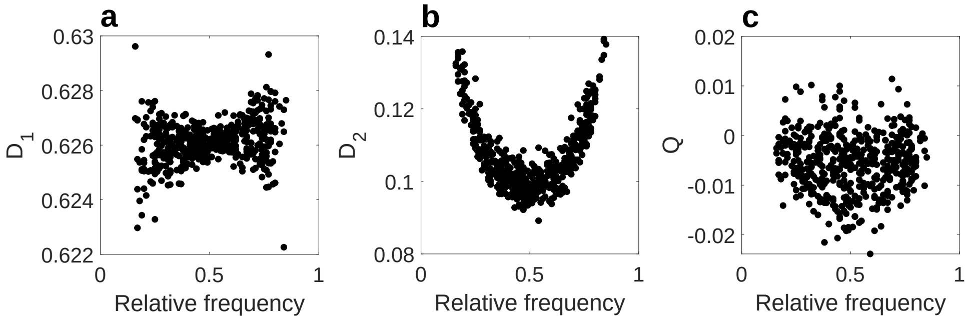

Supplementary Figure 1: **Bias of the three EPI measures due to the relative frequency of species in simulated random data.** The random data matrix $X^{N \times M}$ of $N = 500$ species and $M = 100$ samples was generated like so: In each sample, the relative frequency of each species, i.e., the probability p_i of a species i to be present in each sample, was taken from a uniform distribution $\mathbb{U}(0.2, 0.8)$ and the abundance of each species was taken from a uniform distribution $\mathbb{U}(0, 1)$. Different samples were generated independently without underlying relationships between the species. Nevertheless, there is a bias of EPI values due to the relative frequency of the species. **a**, The variance of D_1^i is greater for species with high or low relative frequency. **b**, There is a parabolic relationship between D_2^i and the relative frequency. **c**, The variance of Q is larger for the species in the middle of the relative frequency spectrum. In addition, the negative values of Q are caused because in the process of calculating modularity, we do not account for the fact that self loops are forbidden. While it is possible to add a correction term to account for self loops, we chose not to do it in order to keep the modularity definition consistent with other sources.

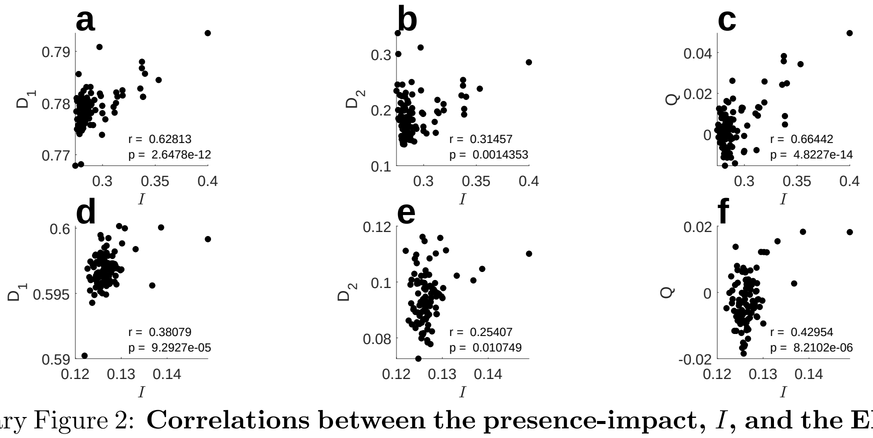

Supplementary Figure 2: **Correlations between the presence-impact, I, and the EPI measures in simulations. a-c**, Correlation between the presence-impact, I, and EPI measures, D_1, D_2 and Q, for interaction-strength-based keystones, created by drawing a boosting parameter K^i for each species i from a log-normal distribution with parameters $\mu = 0.5$ and $\sigma = 1$. The corresponding Pearson correlations and p values are written in the figure. **d-f**, Similar to (**a-c**) for Structure-based keystone created by initializing the interaction network with a scale-free BA topology with $n_0 = 3$ and $n = 2$. The interaction strengths were scaled by 0.5 to ensure the simulations' stability.

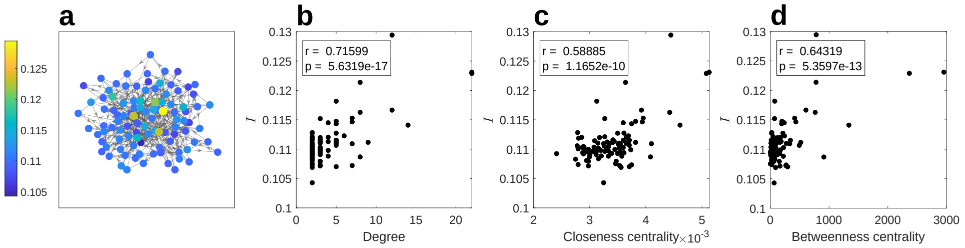

Supplementary Figure 3: **Relationship between the presence-impact I and the underlying interaction network.** **a**, We perform perturbation experiments on a system characterized by an underlying interaction network that is a scale-free BA network. The nodes which represent the species are colored by their presence-impact I. The centralized species are with the largest presence-impact. **b-d**, We calculate the correlation between the presence-impact and different centrality measures, Degree, Closeness centrality, and Betweenness centrality. The corresponding Pearson correlations and p values are written in the figures and show a significant correlation between all three measures.

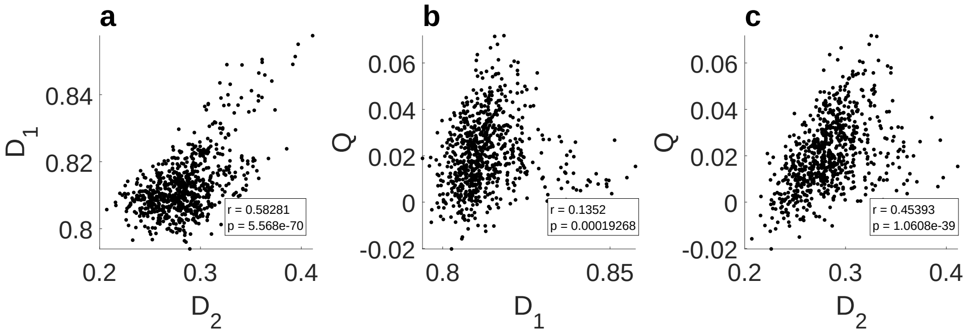

Supplementary Figure 4: **Correlations between the EPI measures for real gut high-throughput sequencing.** **a**, Correlation between D_1 and D_2. **b**, Correlation between Q and D_1. **c**, Correlation between Q and D_2. The dots represent the EPI values of all $N = 1000$ top abundant species. The corresponding Pearson correlations and p values are written in the figures. The relatively small correlation between Q and D_1 is may be related to the relative frequency bias of D_1.

To analyze the relation between the presence-impact of a species and its abundance in real microbiome datasets, we have plotted the D_2 values versus the mean abundance of all species in all the datasets we analyzed from the HMP repository. The following figure shows the results (Supplementary Figure 26 in the revised manuscript) as well as the associated Pearson correlation coefficients.

Supplementary Figure 26 demonstrates rich results. In some body sites, the species' EPI value seems to be correlated with their abundances (*Left antecubital fossa* and *right antecubital fossa*, panels **g** and **l**). On other body sites, while the overall correlation is low, the top abundant species are also among those with high EPI values (for example in the stool samples, panels **a** and **c**). Yet, in most cases, the EPI value seems to be independent upon the abundance, and the species with the highest EPI values, i.e., the keystone candidates, are not among the most abundant species.

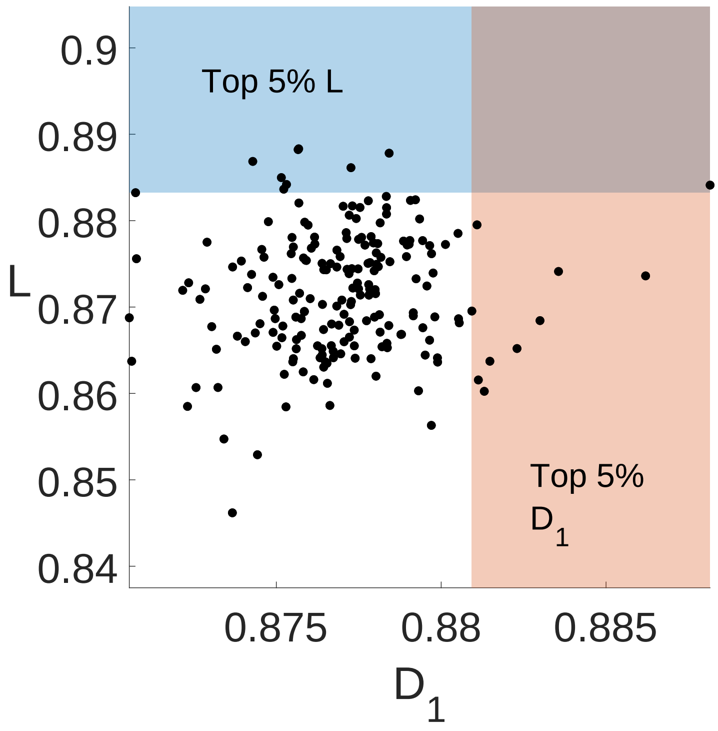

Supplementary Figure 5: **Longitudinal EPI of shuffled data.** Same as Fig. 5**b** in the main text but for shuffled data, where the abundance of each species was randomly assigned between the samples, persevering the relative frequency. In this case, there is no relationship between the EPI of species calculated from cross-sectional data and the presence-impact calculated from longitudinal data. The Pearson coefficient is equal to $r = 0.19$ with $p = 0.0057$. Fisher's test of the overlapping candidates also gives a non-significant result, with $p = 0.2765$. The shuffling was done in a weighted manner, preserving the total number of observed species in each sample.

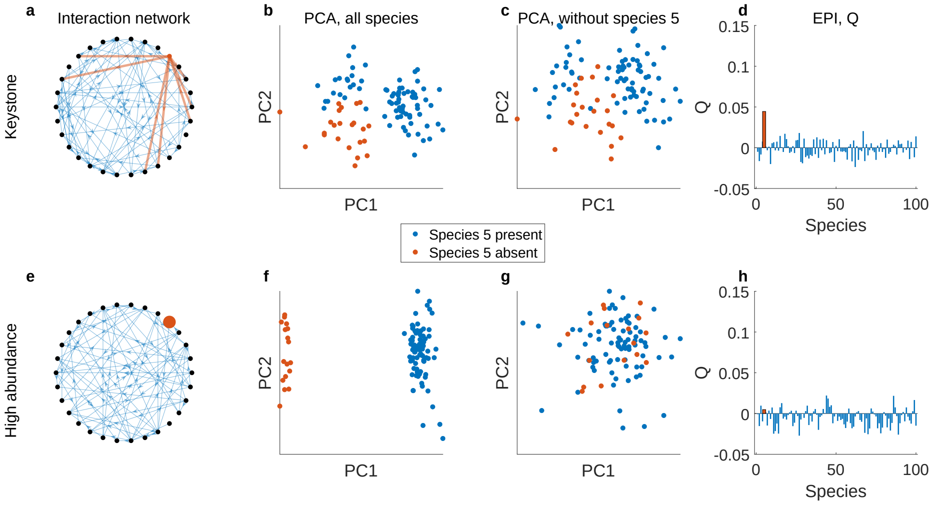

Supplementary Figure 6: **Comparison between the effect of keystone species and high-abundance species on the EPI method and PCA.** **a**, Interaction network with a keystone species (species number 5) marked in red. The interactions of the species (red lines) were multiplied by a constant to make them larger than the average species. **b**, PCA plot of 100 samples generated with the interaction network in a. Samples where the keystone species is present are marked by the red color. The separation between the samples is evident. **c**, PCA plot of the samples, with species number 5 removed from the data. The separation is still evident as the effect of the species is still present, even without considering the abundance of the keystone itself. **d**, The modularity EPI value, Q, for the different species. Species number 5 is significantly larger. **e**, Same as a, but for a non-keystone species. Instead, species number 5 has only elevated abundance levels. **f**, Even though the effect of the species, in this case, is not significantly higher than an average species, the PCA plot is divided into the two types of samples, as the abundance of the species itself is enough to alter the abundance of the rest of the species due to normalization effects. **g**, After removing species number 5, the samples are indistinguishable in the PCA space. **h**, The modularity value, Q, does not indicate a keystone species in this case.

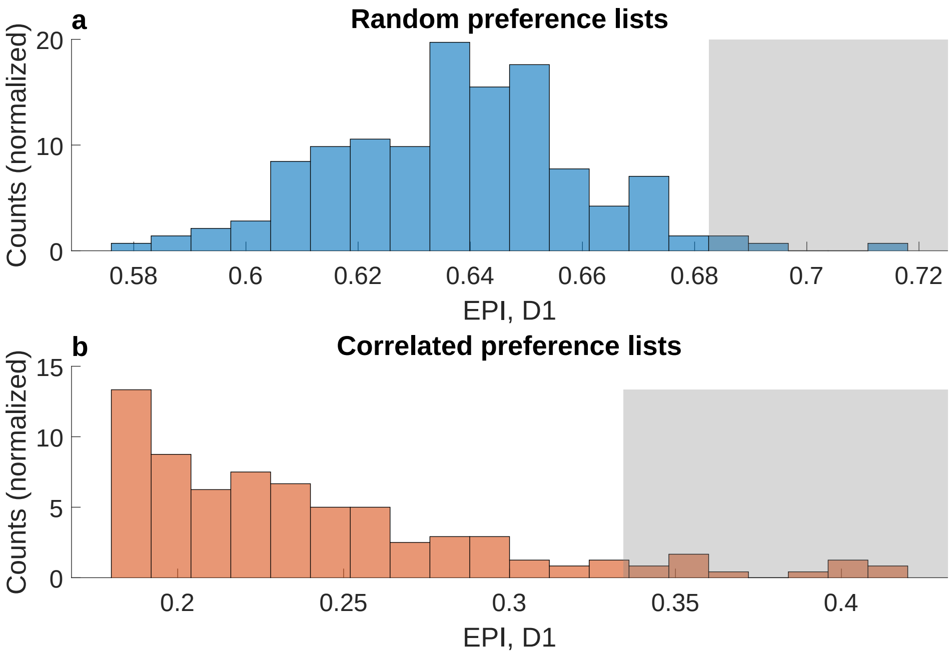

Supplementary Figure 7: **Detecting keystone species in the stable marriage model using the EPI method.** We model microbial samples using the stable marriage model as described in Ref. [23]. **a**, When the species and nutrients have random preference lists, the distribution of EPI values has a major peak with a few species with high EPI value due to random fluctuations. **b**, When the preference lists are correlated, the removal of a single species has a higher probability to resolve conflicts and therefore to cause a significant shift in the balance of the other species. Such a frustrated system will be detected as a large tail in the EPI distribution of the species. Here, the correlation factor $x = 0.025$ (as described in the Supplementary Information of [23]), corresponds to an average Spearman correlation value between the preference lists of 0.96.

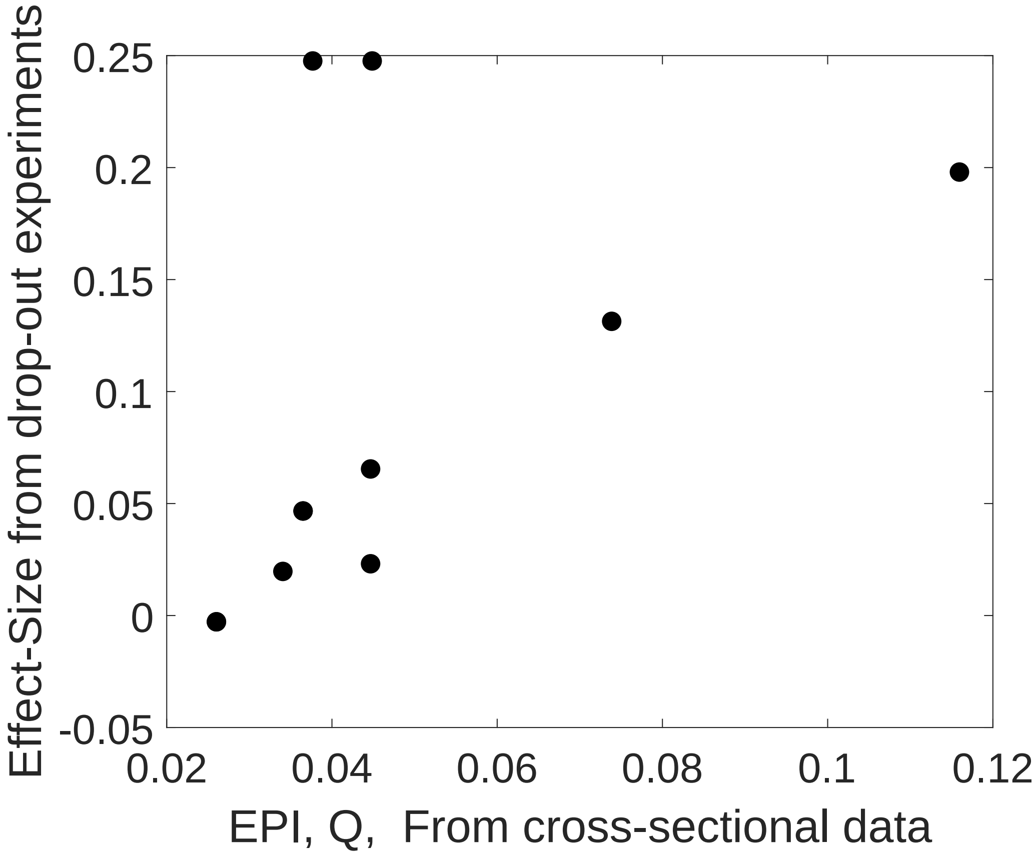

Supplementary Figure 8: **Effect size of perturbation experiments versus EPI values from cross-sectional data from two independent studies.** We compare the results of two independent studies of the microbiome of *Arabidopsis* leaf. In the first, Bai, Vorholt et al [30] collected microbial samples of the leaf of wild Arabidopsis and categorized the taxa into operational units they called At-LSPHERE. In the second study by Carlström, Vorholt, et al [29], the authors performed perturbation experiments on synthetic Arabidopsis microbial environments and quantified keystone species by their effect-size. We have compared the effect size from the perturbation, single-strain drop-out experiments with the EPI Q values of the cross-sectional samples, to every shared taxon that was present in both data sets ($n = 9$). The Pearson coefficient of this analysis is $r = 0.7926$ with $p = 0.0036$.

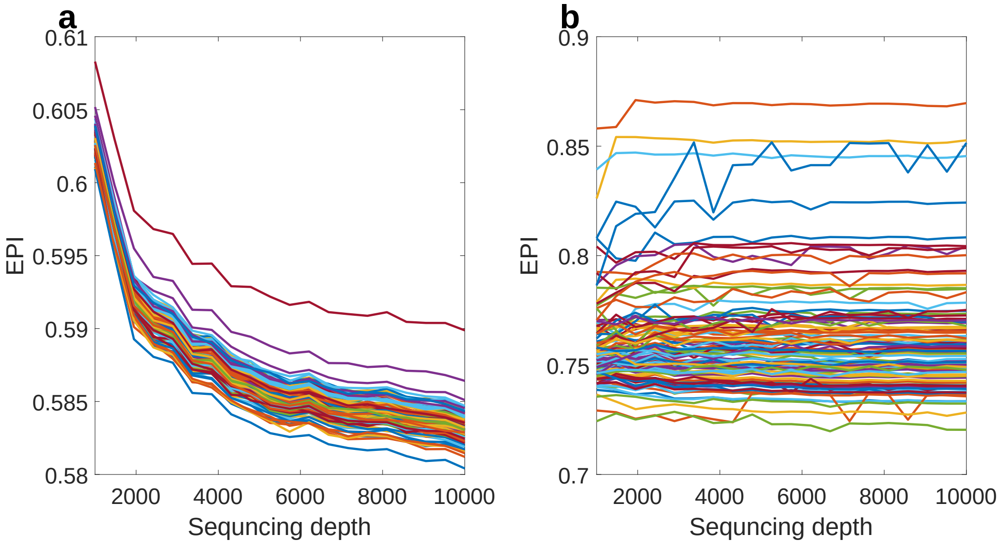

Supplementary Figure 9: **EPI, D_1 values as a function of sequencing depth for simulated and real data a**, Simulated GLV dynamics. **b**, Gut microbiome from the HMP. Species with large EPI have a tendency to preserve their high ranking even when the sequencing depth is increased.

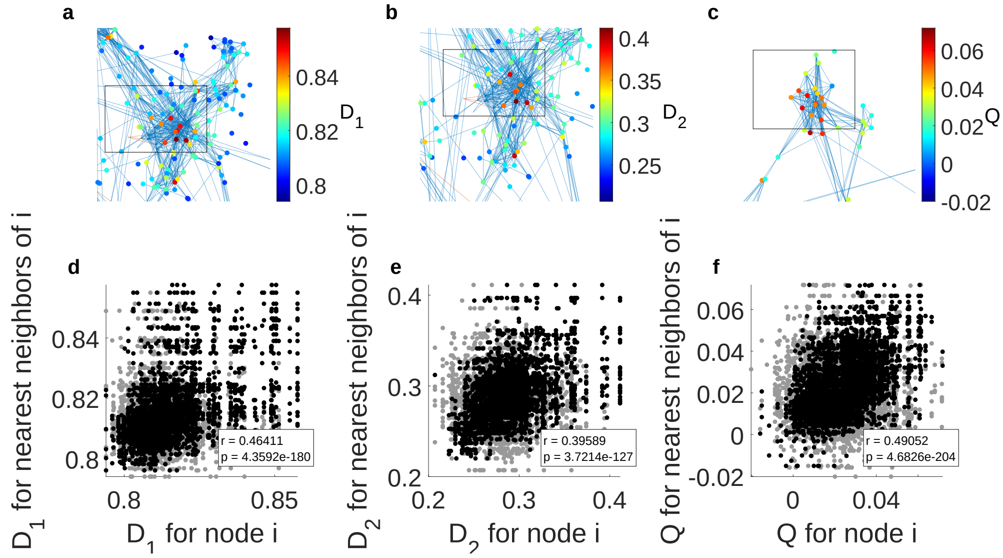

Supplementary Figure 10: **Keystone modules in the presence-absence co-occurrence network with the Jaccard similarity measure.** Similar to Figure 6 but with the Jaccard similarity measure instead of the Mutual Information measure. Detail of the co-occurrence network of species based on the presence-absence data. Edges represent the top 25 percentile of normalized Jaccard similarity values calculated between all species pairs. The edges were colored according to the Pearson correlation, where blue (red) indicates positive (negative) correlations. Each node (species) in the network is colored by its EPI D_1 value. The black rectangle highlights an example of a typical group of highly correlated species which large EPI values. All the species in the rectangle are of the genus *Bacteroides*. **b**, Similar to (**a**) for D_2. All the splecies in the rectangle are of the genus *Bacteroides*. **c**, Similar to (**a**) for Q. All the species in the rectangle are of the genus *Faecalibacterium*. **d**, We statistically study the relation between the network structure and the EPI values of the nodes by calculating the correlation between the EPI D_1 values of nearest neighbors species (black dots). The grey dots represent the same values after randomly shuffling the EPI values among the species. Pearson correlation scores and associated p values are presented in the figure. **e-f**, Similar to (**d**) for D_2 and Q.

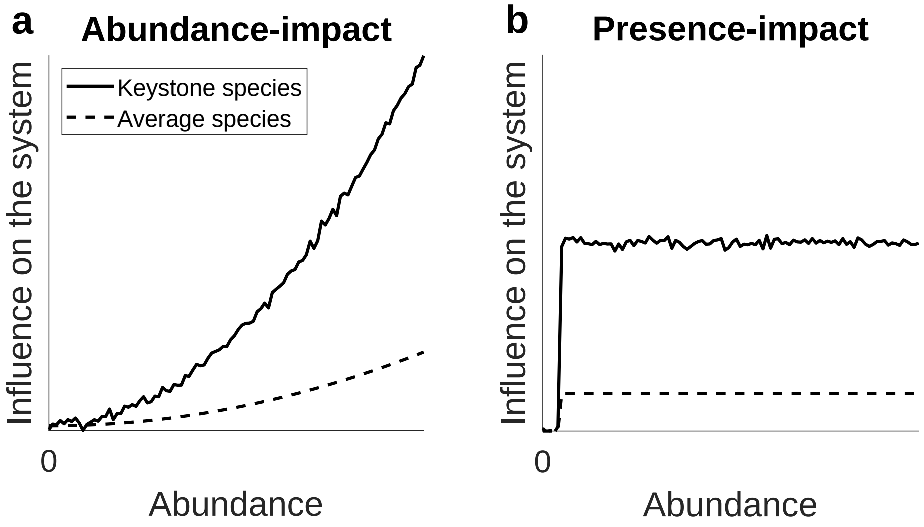

Supplementary Figure 11: **Schematic difference between abundance-impact and presence-impact of keystone taxa.** **a**, Abundance-impact. As the abundance of a species in a system increases, its influence on the system also increases. Keystone taxa are then defined as having an especially strong influence on the system as a function of their abundance, compared to regular taxa. **b**, Presence-impact. When the taxon is introduced, it causes a significant disturbance to the system even though its abundance is small. Once the species is present, the dependence of the influence on the abundance of the species is negligible. Keystone taxa in this context have an especially large influence on the system as soon as they are introduced into it.

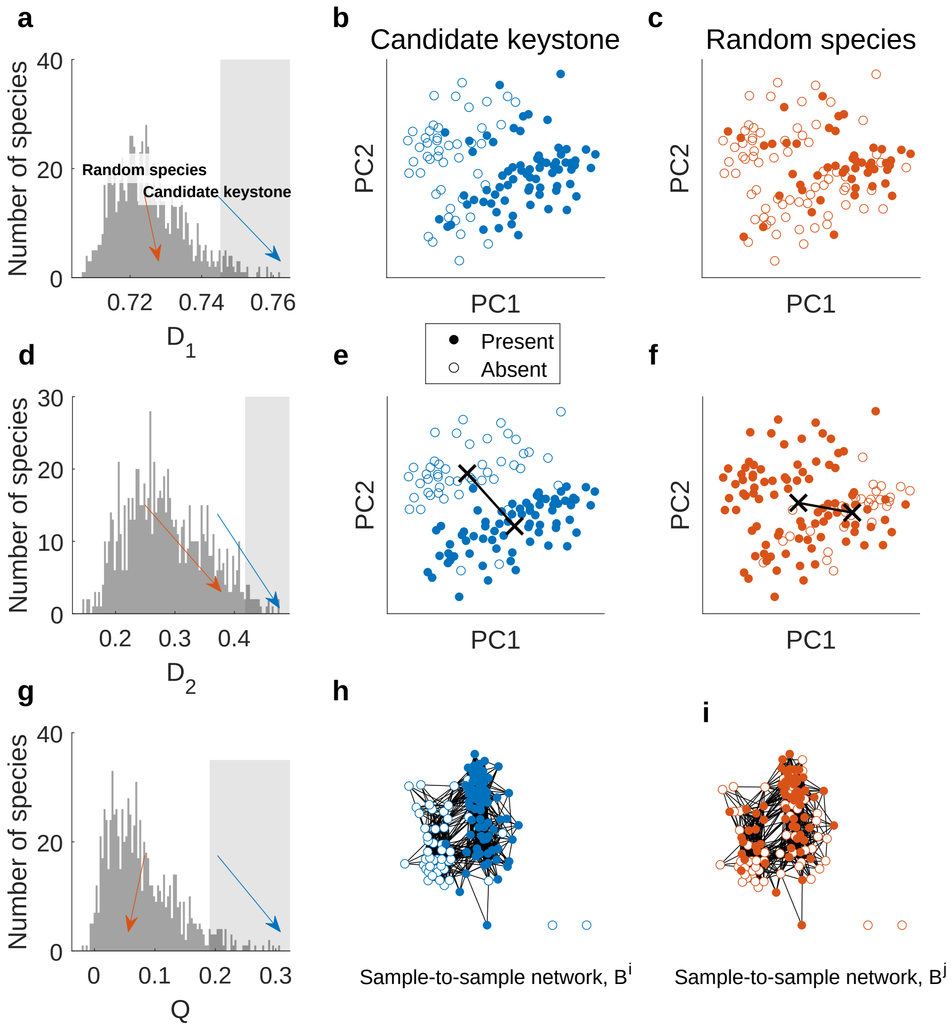

Supplementary Figure 12: **EPI of real high-throughput sequencing.** Same as Fig. 4 for the *Tongue dorsum* dataset from the HMP

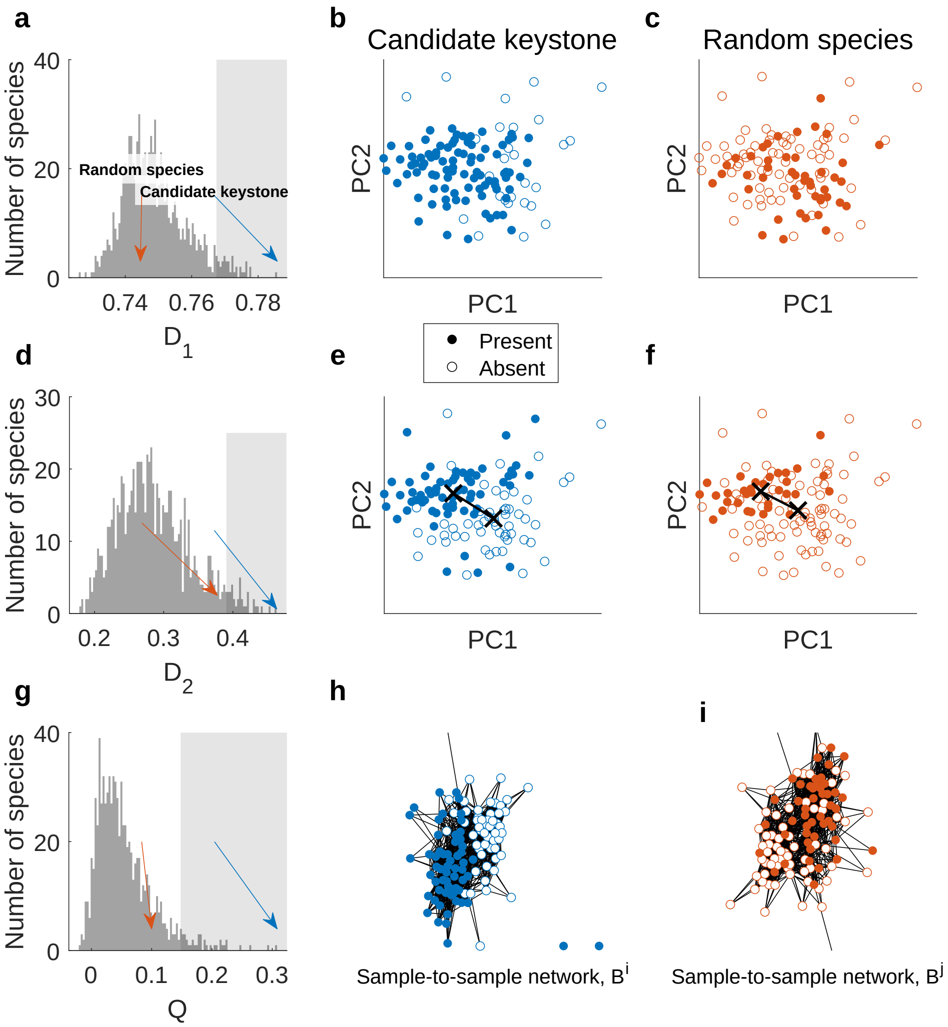

Supplementary Figure 13: **EPI of real high-throughput sequencing.** Same as Fig. 4 for the *Throat* dataset from the HMP. **a**, Distribution of the EPI D_1 values of all $N = 1000$ top abundant species. The grey area marks the EPI values greater than two standard deviations from the mean. Blue and red arrows mark the EPI values of a candidate keystone, i, and a random species, j, respectively. **b**, PCoA visualization of keystone associated abundance profiles S_k^i. Filled dots represent samples where the species is present, empty circles represent samples where the species is absent. The samples are naturally separated by the absent/presence of the keystone species into two types. **c**, Similar to (**b**) for the random species j. Here there is no visible separation of the samples into types. **d-f**, Similar to (**a-c**) for the EPI D_2. The black crosses mark the mean of the groups. **g**, Similar to (**a**) for the modularity EPI measure, Q. **h**, The sample-to-sample correlation network, B^i, associated with the keystone candidate i. Filled (empty) nodes represent samples where the species is present (absent). The natural separation between the nodes into two groups indicates the large modularity value Q^i. **i**, Similar to (**h**) for a random species j. The lack of separation between the groups indicates the low modularity value Q^j of the random species.

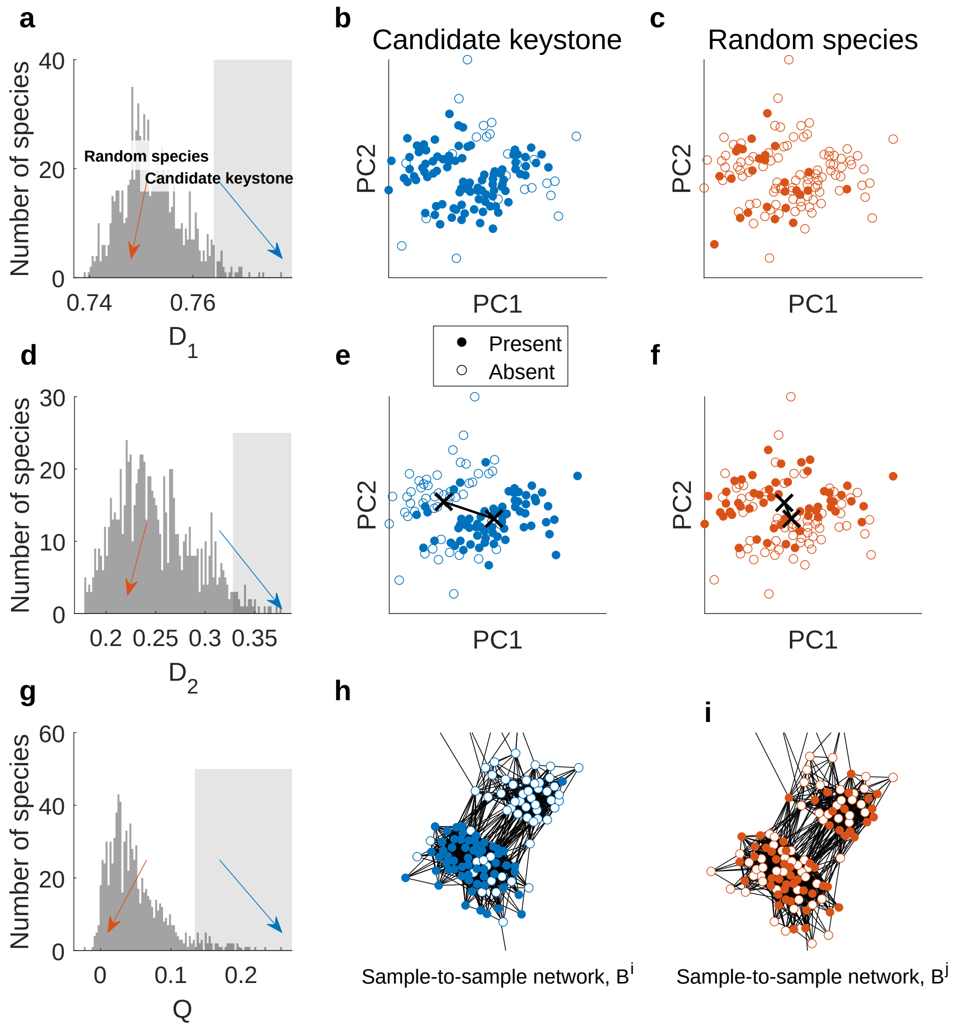

Supplementary Figure 14: **EPI of real high-throughput sequencing.** Same as Fig. 4 for the *Supragingival plaque* dataset from the HMP. **a**, Distribution of the EPI D_1 values of all $N = 1000$ top abundant species. The grey area marks the EPI values greater than two standard deviations from the mean. Blue and red arrows mark the EPI values of a candidate keystone, i, and a random species, j, respectively. **b**, PCoA visualization of keystone associated abundance profiles S^i_k. Filled dots represent samples where the species is present, empty circles represent samples where the species is absent. The samples are naturally separated by the absent/presence of the keystone species into two types. **c**, Similar to (**b**) for the random species j. Here there is no visible separation of the samples into types. **d-f**, Similar to (**a-c**) for the EPI D_2. The black crosses mark the mean of the groups. **g**, Similar to (**a**) for the modularity EPI measure, Q. **h**, The sample-to-sample correlation network, B^i, associated with the keystone candidate i. Filled (empty) nodes represent samples where the species is present (absent). The natural separation between the nodes into two groups indicates the large modularity value Q^i. **i**, Similar to (**h**) for a random species j. The lack of separation between the groups indicates the low modularity value Q^j of the random species.

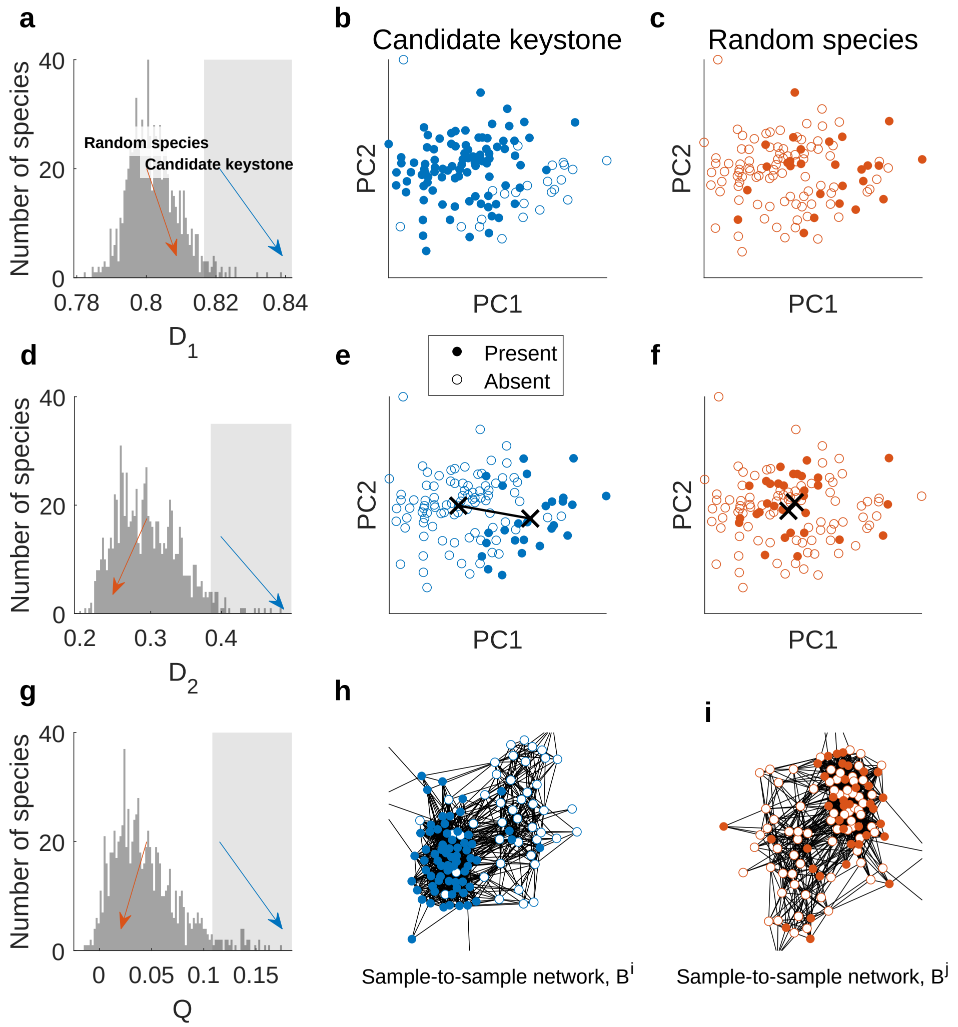

Supplementary Figure 15: **EPI of real high-throughput sequencing.** Same as Fig. 4 for the *Subgingival plaque* dataset from the HMP. **a**, Distribution of the EPI D_1 values of all $N = 1000$ top abundant species. The grey area marks the EPI values greater than two standard deviations from the mean. Blue and red arrows mark the EPI values of a candidate keystone, i, and a random species, j, respectively. **b**, PCoA visualization of keystone associated abundance profiles S_k^i. Filled dots represent samples where the species is present, empty circles represent samples where the species is absent. The samples are naturally separated by the absent/presence of the keystone species into two types. **c**, Similar to (**b**) for the random species j. Here there is no visible separation of the samples into types. **d-f**, Similar to (**a-c**) for the EPI D_2. The black crosses mark the mean of the groups. **g**, Similar to (**a**) for the modularity EPI measure, Q. **h**, The sample-to-sample correlation network, B^i, associated with the keystone candidate i. Filled (empty) nodes represent samples where the species is present (absent). The natural separation between the nodes into two groups indicates the large modularity value Q^i. **i**, Similar to (**h**) for a random species j. The lack of separation between the groups indicates the low modularity value Q^j of the random species.

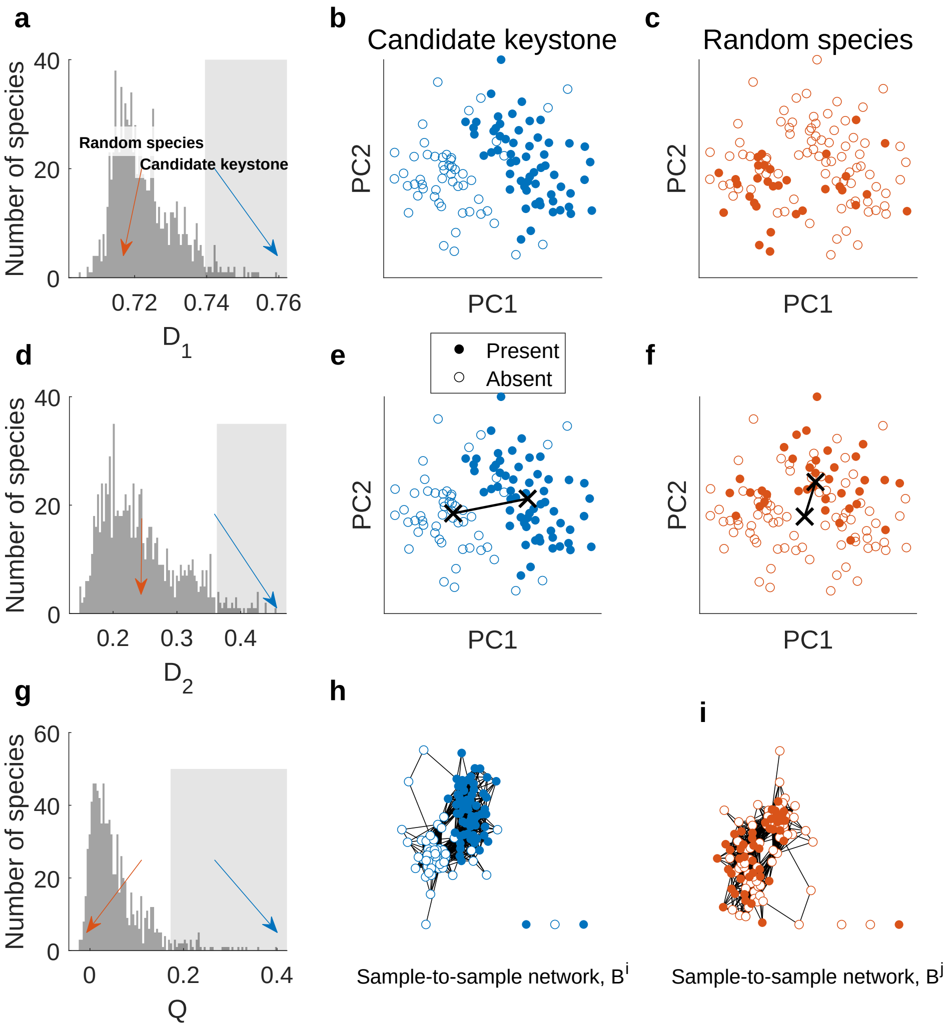

Supplementary Figure 16: **EPI of real high-throughput sequencing.** Same as Fig. 4 for the *Saliva* dataset from the HMP. **a**, Distribution of the EPI D_1 values of all $N = 1000$ top abundant species. The grey area marks the EPI values greater than two standard deviations from the mean. Blue and red arrows mark the EPI values of a candidate keystone, i, and a random species, j, respectively. **b**, PCoA visualization of keystone associated abundance profiles S_k^i. Filled dots represent samples where the species is present, empty circles represent samples where the species is absent. The samples are naturally separated by the absent/presence of the keystone species into two types. **c**, Similar to (**b**) for the random species j. Here there is no visible separation of the samples into types. **d-f**, Similar to (**a-c**) for the EPI D_2. The black crosses mark the mean of the groups. **g**, Similar to (**a**) for the modularity EPI measure, Q. **h**, The sample-to-sample correlation network, B^i, associated with the keystone candidate i. Filled (empty) nodes represent samples where the species is present (absent). The natural separation between the nodes into two groups indicates the large modularity value Q^i. **i**, Similar to (**h**) for a random species j. The lack of separation between the groups indicates the low modularity value Q^j of the random species.

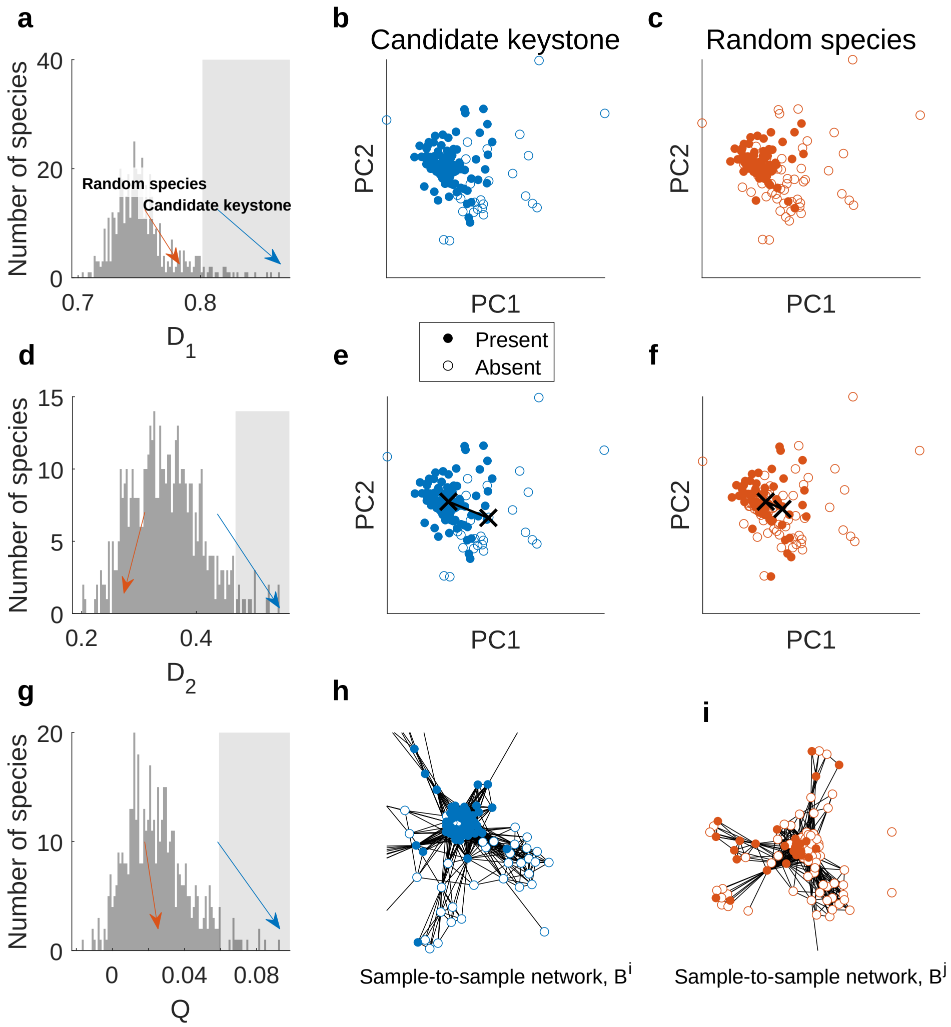

Supplementary Figure 17: **EPI of real high-throughput sequencing.** Same as Fig. 4 for the *Right Retroauricular crease* dataset from the HMP. **a**, Distribution of the EPI D_1 values of all $N = 1000$ top abundant species. The grey area marks the EPI values greater than two standard deviations from the mean. Blue and red arrows mark the EPI values of a candidate keystone, i, and a random species, j, respectively. **b**, PCoA visualization of keystone associated abundance profiles S_k^i. Filled dots represent samples where the species is present, empty circles represent samples where the species is absent. The samples are naturally separated by the absent/presence of the keystone species into two types. **c**, Similar to (**b**) for the random species j. Here there is no visible separation of the samples into types. **d-f**, Similar to (**a-c**) for the EPI D_2. The black crosses mark the mean of the groups. **g**, Similar to (**a**) for the modularity EPI measure, Q. **h**, The sample-to-sample correlation network, B^i, associated with the keystone candidate i. Filled (empty) nodes represent samples where the species is present (absent). The natural separation between the nodes into two groups indicates the large modularity value Q^i. **i**, Similar to (**h**) for a random species j. The lack of separation between the groups indicates the low modularity value Q^j of the random species.

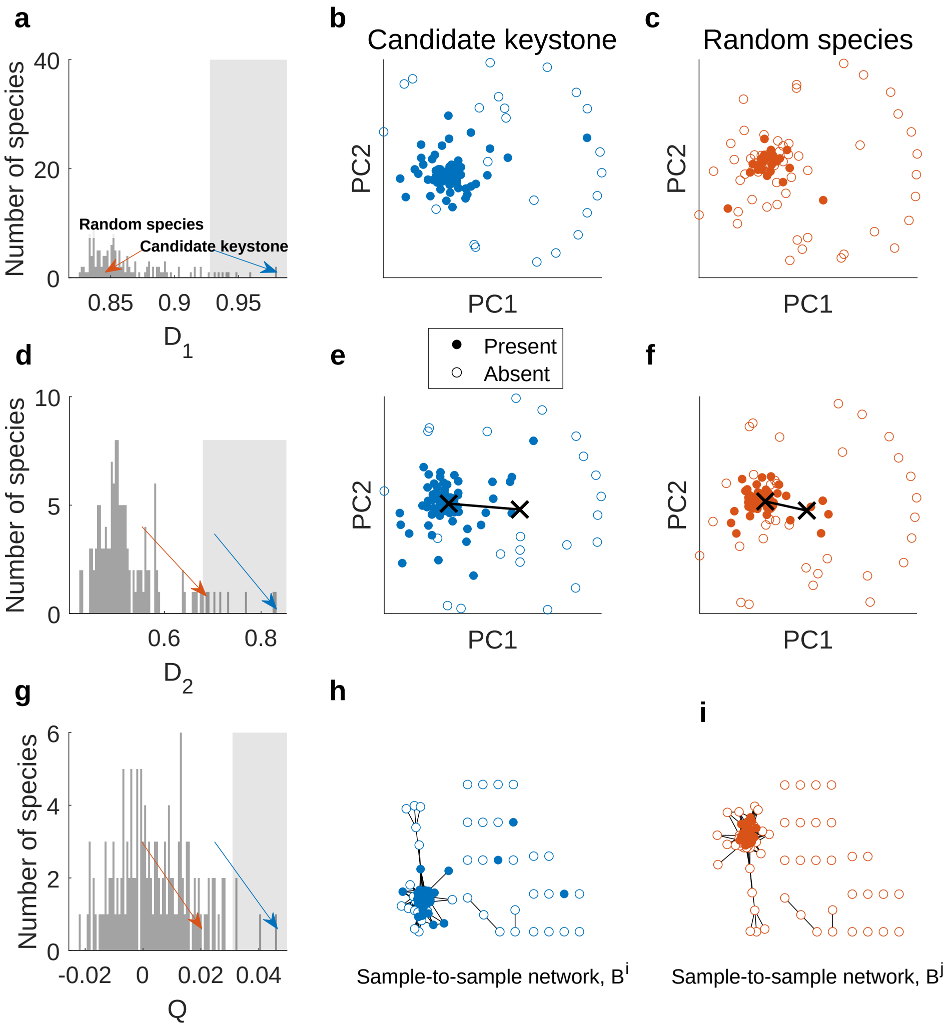

Supplementary Figure 18: **EPI of real high-throughput sequencing.** Same as Fig. 4 for the *Right Antecubital fossa* dataset from the HMP. **a**, Distribution of the EPI D_1 values of all $N = 1000$ top abundant species. The grey area marks the EPI values greater than two standard deviations from the mean. Blue and red arrows mark the EPI values of a candidate keystone, i, and a random species, j, respectively. **b**, PCoA visualization of keystone associated abundance profiles S_k^i. Filled dots represent samples where the species is present, empty circles represent samples where the species is absent. The samples are naturally separated by the absent/presence of the keystone species into two types. **c**, Similar to (**b**) for the random species j. Here there is no visible separation of the samples into types. **d-f**, Similar to (**a-c**) for the EPI D_2. The black crosses mark the mean of the groups. **g**, Similar to (**a**) for the modularity EPI measure, Q. **h**, The sample-to-sample correlation network, B^i, associated with the keystone candidate i. Filled (empty) nodes represent samples where the species is present (absent). The natural separation between the nodes into two groups indicates the large modularity value Q^i. **i**, Similar to (**h**) for a random species j. The lack of separation between the groups indicates the low modularity value Q^j of the random species.

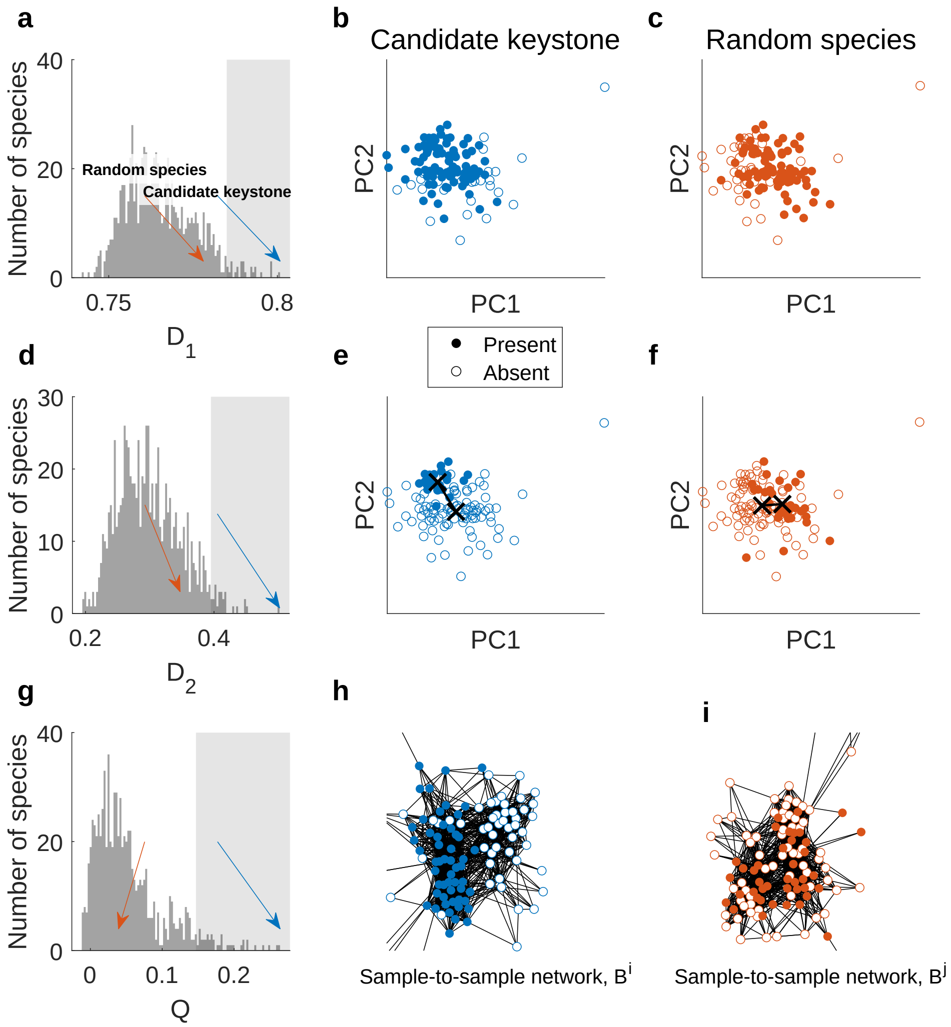

Supplementary Figure 19: **EPI of real high-throughput sequencing.** Same as Fig. 4 for the *Palatine Tonsils* dataset from the HMP. **a**, Distribution of the EPI D_1 values of all $N = 1000$ top abundant species. The grey area marks the EPI values greater than two standard deviations from the mean. Blue and red arrows mark the EPI values of a candidate keystone, i, and a random species, j, respectively. **b**, PCoA visualization of keystone associated abundance profiles S_k^i. Filled dots represent samples where the species is present, empty circles represent samples where the species is absent. The samples are naturally separated by the absent/presence of the keystone species into two types. **c**, Similar to (**b**) for the random species j. Here there is no visible separation of the samples into types. **d-f**, Similar to (**a-c**) for the EPI D_2. The black crosses mark the mean of the groups. **g**, Similar to (**a**) for the modularity EPI measure, Q. **h**, The sample-to-sample correlation network, B^i, associated with the keystone candidate i. Filled (empty) nodes represent samples where the species is present (absent). The natural separation between the nodes into two groups indicates the large modularity value Q^i. **i**, Similar to (**h**) for a random species j. The lack of separation between the groups indicates the low modularity value Q^j of the random species.

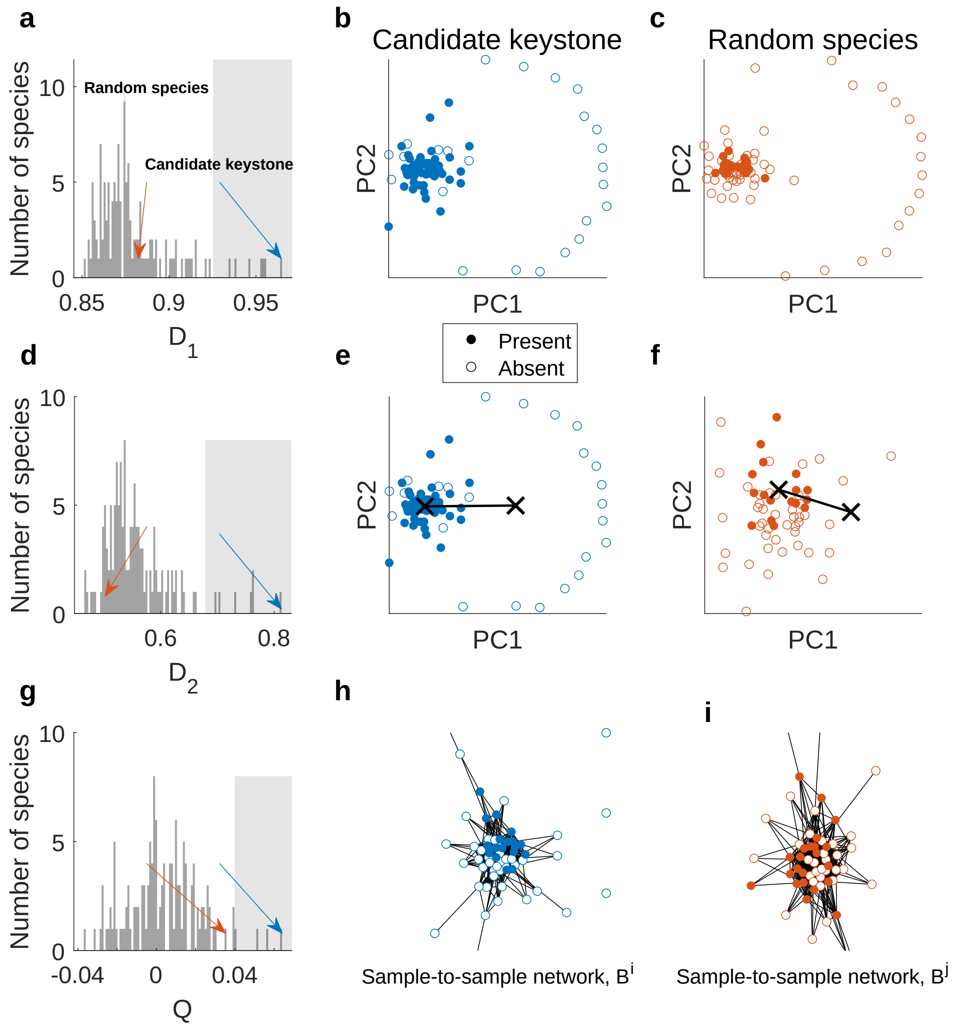

Supplementary Figure 20: **EPI of real high-throughput sequencing.** Same as Fig. 4 for the *Left Antecubital mfossa* dataset from the HMP. **a**, Distribution of the EPI D_1 values of all $N = 1000$ top abundant species. The grey area marks the EPI values greater than two standard deviations from the mean. Blue and red arrows mark the EPI values of a candidate keystone, i, and a random species, j, respectively. **b**, PCoA visualization of keystone associated abundance profiles S_k^i. Filled dots represent samples where the species is present, empty circles represent samples where the species is absent. The samples are naturally separated by the absent/presence of the keystone species into two types. **c**, Similar to (**b**) for the random species j. Here there is no visible separation of the samples into types. **d-f**, Similar to (**a-c**) for the EPI D_2. The black crosses mark the mean of the groups. **g**, Similar to (**a**) for the modularity EPI measure, Q. **h**, The sample-to-sample correlation network, B^i, associated with the keystone candidate i. Filled (empty) nodes represent samples where the species is present (absent). The natural separation between the nodes into two groups indicates the large modularity value Q^i. **i**, Similar to (**h**) for a random species j. The lack of separation between the groups indicates the low modularity value Q^j of the random species.

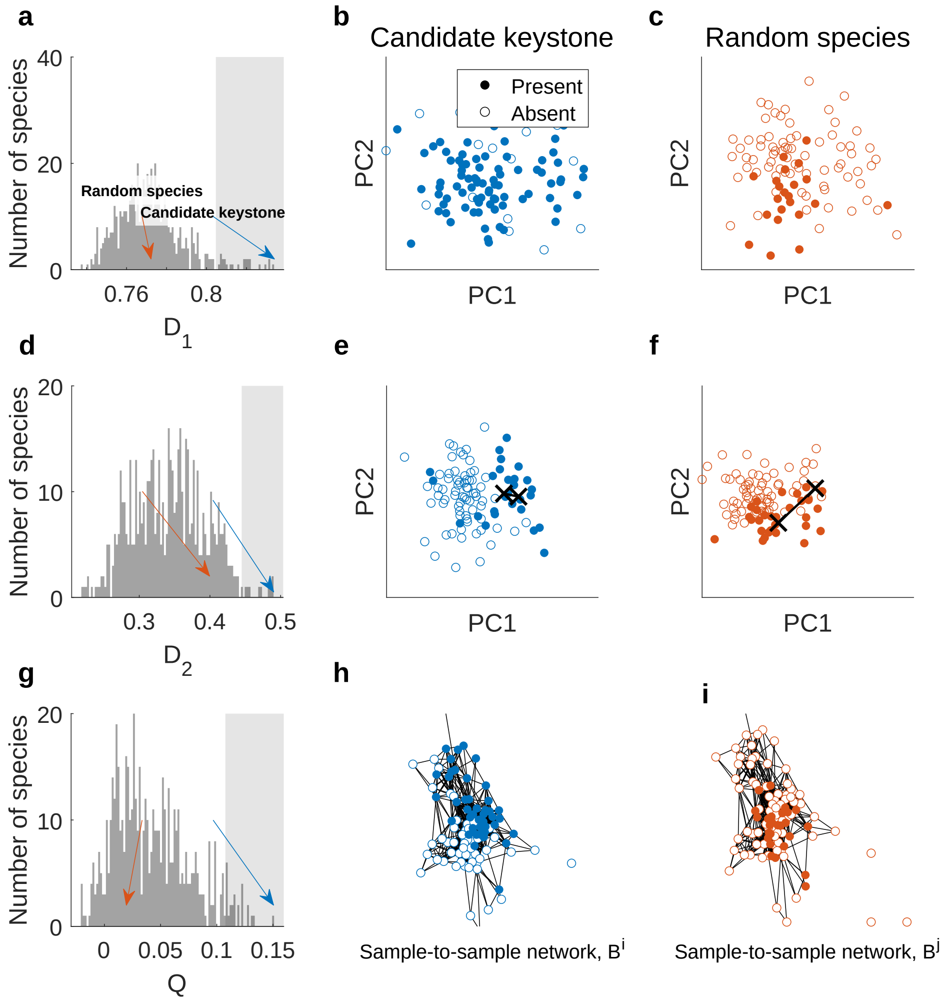

Supplementary Figure 21: **EPI of real high-throughput sequencing.** Same as Fig. 4 for the *Anterior nares* dataset from the HMP. **a**, Distribution of the EPI D_1 values of all $N = 1000$ top abundant species. The grey area marks the EPI values greater than two standard deviations from the mean. Blue and red arrows mark the EPI values of a candidate keystone, i, and a random species, j, respectively. **b**, PCoA visualization of keystone associated abundance profiles S_k^i. Filled dots represent samples where the species is present, empty circles represent samples where the species is absent. The samples are naturally separated by the absent/presence of the keystone species into two types. **c**, Similar to (**b**) for the random species j. Here there is no visible separation of the samples into types. **d-f**, Similar to (**a-c**) for the EPI D_2. The black crosses mark the mean of the groups. **g**, Similar to (**a**) for the modularity EPI measure, Q. **h**, The sample-to-sample correlation network, B^i, associated with the keystone candidate i. Filled (empty) nodes represent samples where the species is present (absent). The natural separation between the nodes into two groups indicates the large modularity value Q^i. **i**, Similar to (**h**) for a random species j. The lack of separation between the groups indicates the low modularity value Q^j of the random species.

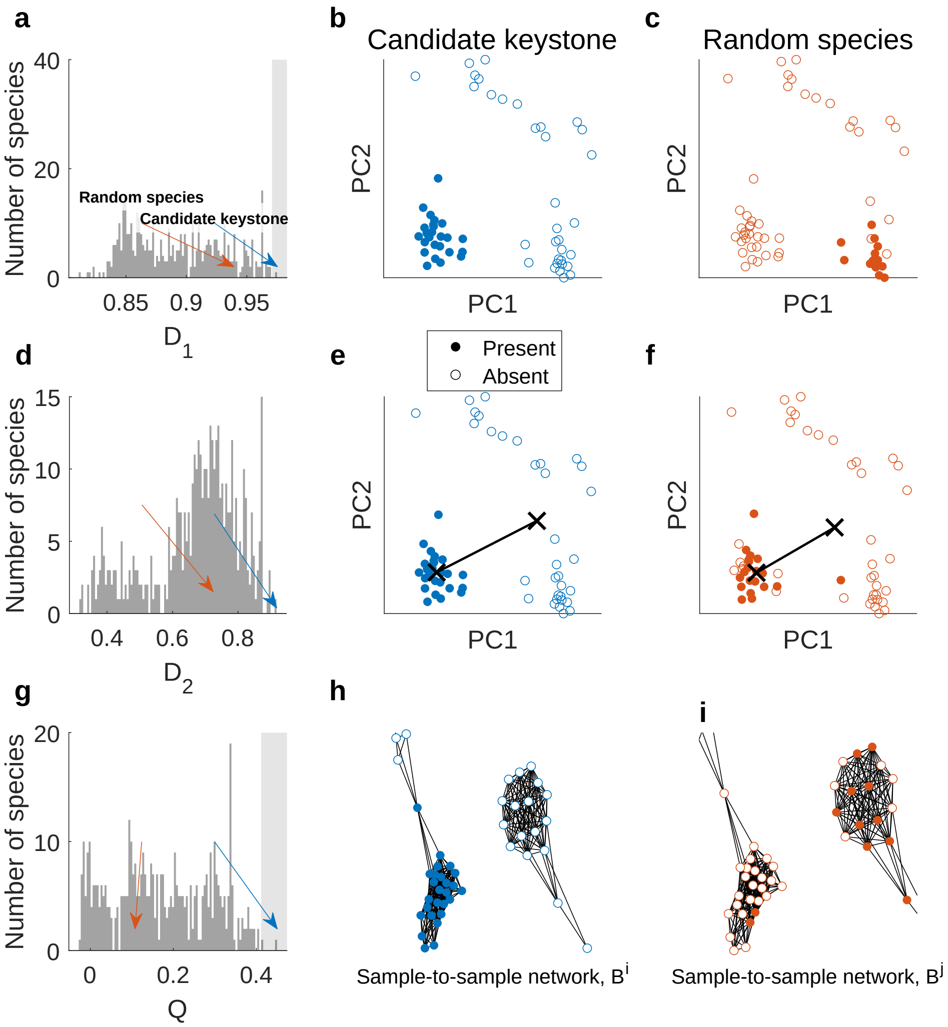

Supplementary Figure 22: **EPI of real high-throughput sequencing.** Same as Fig. 4 for the *Mid vagina* dataset from the HMP. **a**, Distribution of the EPI D_1 values of all $N = 1000$ top abundant species. The grey area marks the EPI values greater than two standard deviations from the mean. Blue and red arrows mark the EPI values of a candidate keystone, i, and a random species, j, respectively. **b**, PCoA visualization of keystone associated abundance profiles S_k^i. Filled dots represent samples where the species is present, empty circles represent samples where the species is absent. The samples are naturally separated by the absent/presence of the keystone species into two types. **c**, Similar to (**b**) for the random species j. Here there is no visible separation of the samples into types. **d-f**, Similar to (**a-c**) for the EPI D_2. The black crosses mark the mean of the groups. **g**, Similar to (**a**) for the modularity EPI measure, Q. **h**, The sample-to-sample correlation network, B^i, associated with the keystone candidate i. Filled (empty) nodes represent samples where the species is present (absent). The natural separation between the nodes into two groups indicates the large modularity value Q^i. **i**, Similar to (**h**) for a random species j. The lack of separation between the groups indicates the low modularity value Q^j of the random species.

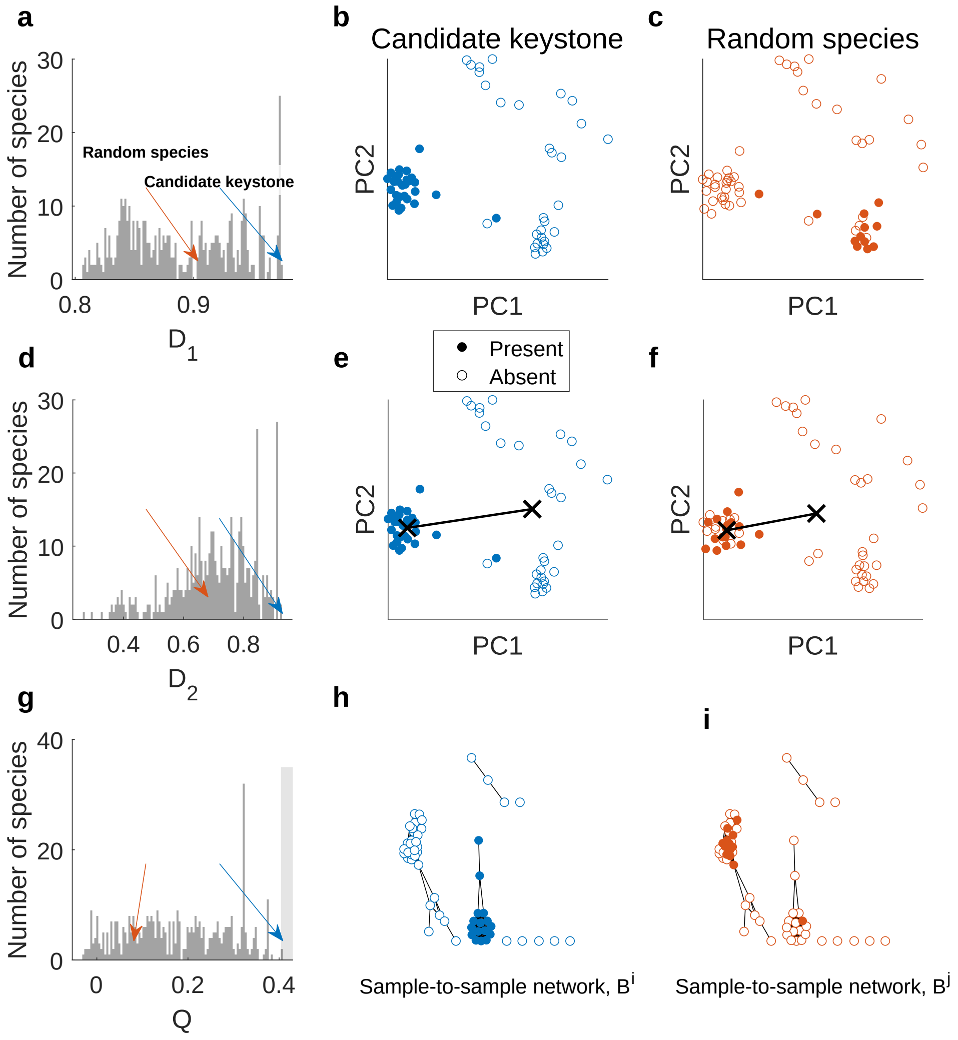

Supplementary Figure 23: **EPI of real high-throughput sequencing.** Same as Fig. 4 for the *Posterior fornix* dataset from the HMP. **a**, Distribution of the EPI D_1 values of all $N = 1000$ top abundant species. The grey area marks the EPI values greater than two standard deviations from the mean. Blue and red arrows mark the EPI values of a candidate keystone, i, and a random species, j, respectively. **b**, PCoA visualization of keystone associated abundance profiles S_k^i. Filled dots represent samples where the species is present, empty circles represent samples where the species is absent. The samples are naturally separated by the absent/presence of the keystone species into two types. **c**, Similar to (**b**) for the random species j. Here there is no visible separation of the samples into types. **d-f**, Similar to (**a-c**) for the EPI D_2. The black crosses mark the mean of the groups. **g**, Similar to (**a**) for the modularity EPI measure, Q. **h**, The sample-to-sample correlation network, B^i, associated with the keystone candidate i. Filled (empty) nodes represent samples where the species is present (absent). The natural separation between the nodes into two groups indicates the large modularity value Q^i. **i**, Similar to (**h**) for a random species j. The lack of separation between the groups indicates the low modularity value Q^j of the random species.

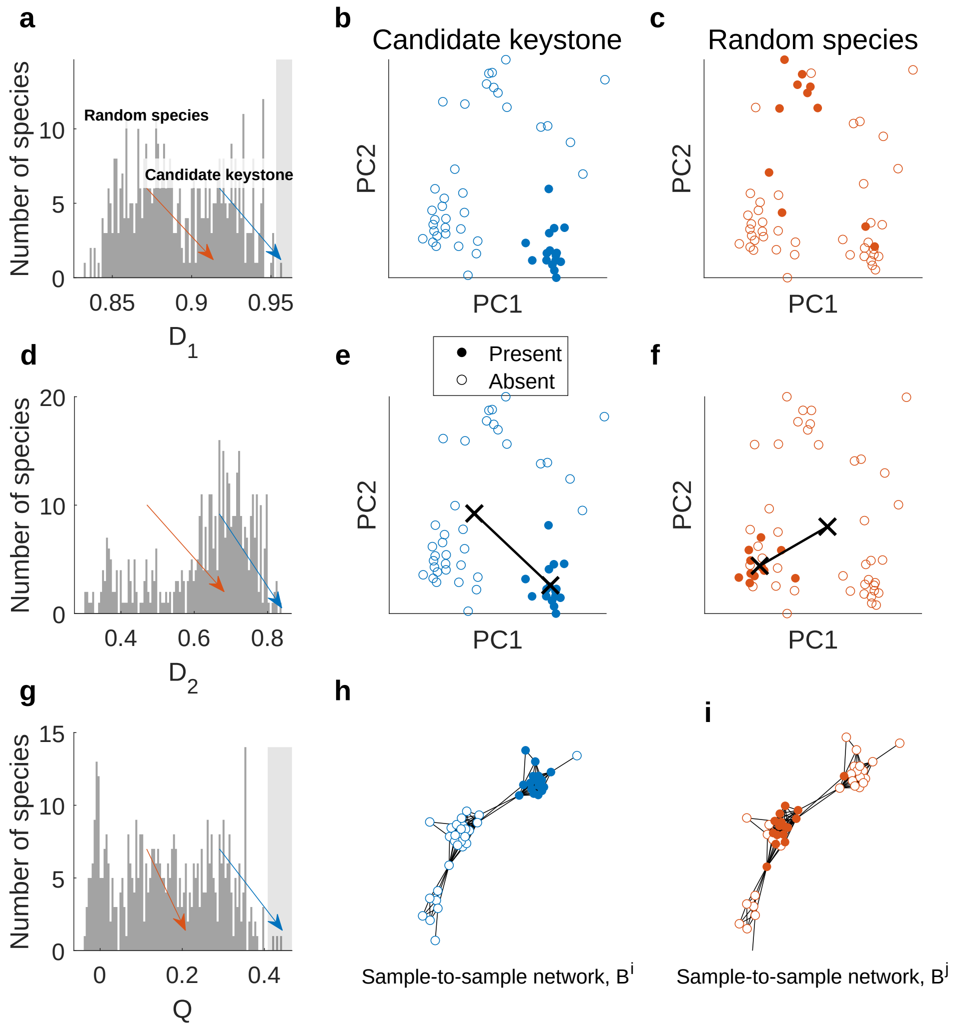

Supplementary Figure 24: **EPI of real high-throughput sequencing.** Same as Fig. 4 for the *Vaginal introitus* dataset from the HMP. **a**, Distribution of the EPI D_1 values of all $N = 1000$ top abundant species. The grey area marks the EPI values greater than two standard deviations from the mean. Blue and red arrows mark the EPI values of a candidate keystone, i, and a random species, j, respectively. **b**, PCoA visualization of keystone associated abundance profiles S_k^i. Filled dots represent samples where the species is present, empty circles represent samples where the species is absent. The samples are naturally separated by the absent/presence of the keystone species into two types. **c**, Similar to (**b**) for the random species j. Here there is no visible separation of the samples into types. **d-f**, Similar to (**a-c**) for the EPI D_2. The black crosses mark the mean of the groups. **g**, Similar to (**a**) for the modularity EPI measure, Q. **h**, The sample-to-sample correlation network, B^i, associated with the keystone candidate i. Filled (empty) nodes represent samples where the species is present (absent). The natural separation between the nodes into two groups indicates the large modularity value Q^i. **i**, Similar to (**h**) for a random species j. The lack of separation between the groups indicates the low modularity value Q^j of the random species.

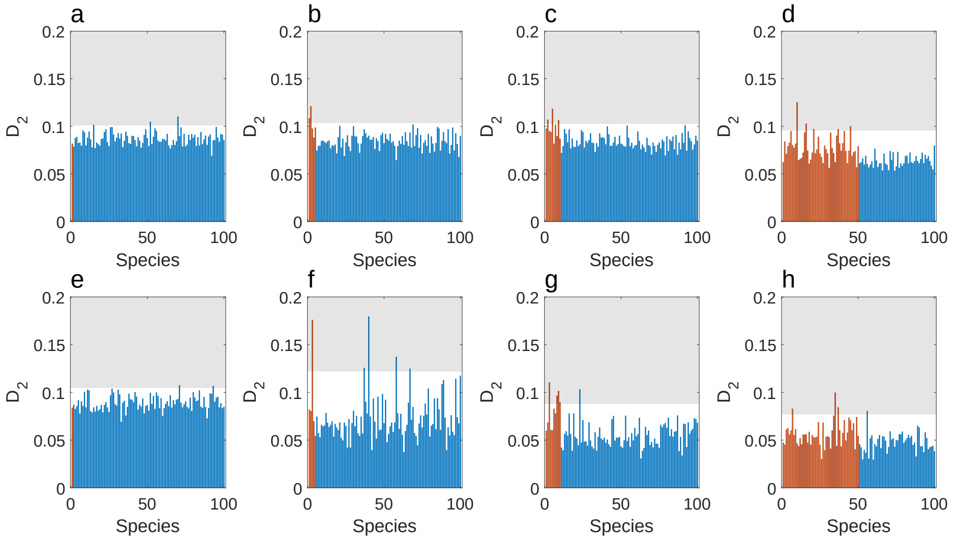

Supplementary Figure 25: **An example of environmental confounding factors affecting keystone candidates' detection in GLV dynamics.** Simulations that mimic the effects of environmental factors in the identification of keystone species. Each sample belongs to environment A or environment B. Both environments have the same underlying interaction network. Species that are colored in red are affected by the type of environment, meaning that their growth rate is randomly increased or decreased by a factor of 2 (top figures) or 50 (bottom figures). Also, their proclivity to be present in the samples is changed randomly from 50% in environment A to 30% or 80% in environment B. Species that are colored in blue are not affected by the environment. When only one species is affected by the environment (**a** and **e**), its likelihood to be detected as a keystone candidate is small, due to the fact that the abundance of all the other species is not spuriously correlated with its presence. Similarly, when many species are affected by the environment (**d** and **h**), their relative importance in the D_2 measure is reduced. When a few species are affected, then the spurious correlations are strong enough to be detected as keystone candidates, as indicated by their D_2 value being larger than two standard deviations from the mean (grey area). Figure **b** and **f** show the distributions when 5 species are affected, and **c** and **g** for 10 species.

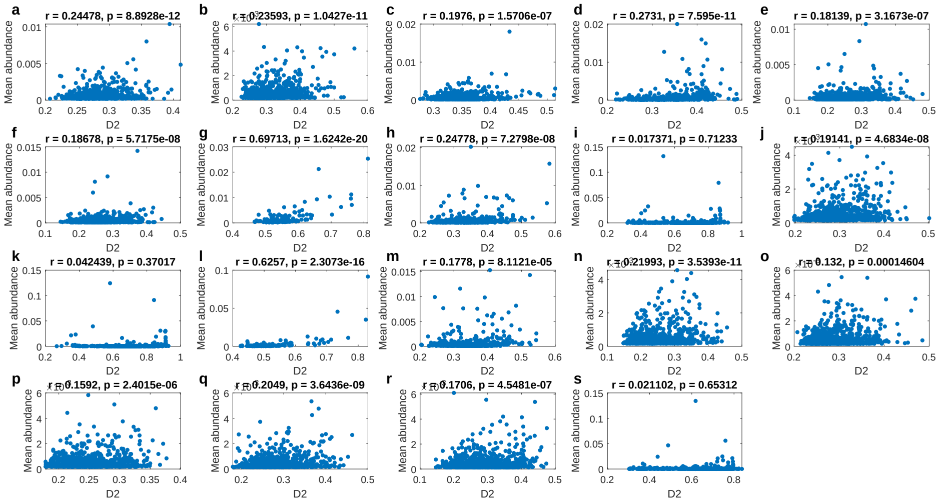

Supplementary Figure 26: D_2 **verus mean abundance for all HMP datasets.** **a**, Stool sample - second visit **b**, Plaque **c**, Stool samples - first visit **d**, Anterior nares **e**, Buccal mucosa **f**, Hard palate **g**, Left Antecubital fossa **h**, Left Retroauricular crease **i**, Mid vagina **j**, Palatine tonsils **k**, Posterior fornix **l**, Right Antecubital fossa **m**, Right Retroauricular crease **n**, Saliva **o**, Subgingival plaque **p**, Supragingival plaque **q**, Throat **r**, Tongue dorsum, **s**, Vaginal introitus. At the top of the figure the Pearson values r is shown together with the p value. While a significant correlation does exists for some data sets, it is clear that high presence impact cannot be explained fully by simply having a large abundance.

Symbol	Definition
N	Number of species
M	Number of samples
t	Time variable in GLV simulations
i, j	Index variables of species
x^i	Abundance of species i in GLV simulations
r^i	Growth rate of species i in GLV simulations
$\mathbf{r}$	$N \times 1$ vector of growth rates of all species in GLV simulations
A	$N \times N$ Interaction matrix in GLV simulations
a_{ij}	Matrix elements of A; the affect of species j on species i
σ_A	Characteristic interaction strengths in GLV simulations
p_{present}	Probability of species to initially be present in the samples in GLV simulations
p_{ER}	Probability of a node being connected to another node in Erdős-Renyi model
K^i	Modification factor of interaction-strength-based keystone for species i
n_0	Number of initial seed nodes in Barabási-Albert network model
n	Number of nodes to be connected in Barabási-Albert model construction process
p_i	Probability of connecting to node i in Barabási-Albert model construction process
d_i	Degree of node i
d	Directionality parameter in Barabási-Albert model
k, k'	Index variables of samples
α, β	Index variables of samples
S_k	Abundance profile of sample k
S_k^i	Abundance profile of sample k, not including species i
S_k^{i*}	Abundance profile of sample k, not including species i, after the removal/addition process
$\langle \cdot \rangle$	Distance function between two samples
I_k^i	Presence-impact of species i based on a single removal/addition experiment from sample k
I^i	Average presence-impact of species i based on removal/addition experiment from all the samples
$\lvert \cdot \rvert$	Size of subset
G^i	The subset of samples where species i is present
$\overline{G^i}$	The subset of samples where species i is absent
D_1^i	EPI of species i based on the average distance between all samples with and without species i present
D_2^i	EPI of species i based on the distance between averages of samples with and without species i present
Q^i	EPI of species i based on the modularity measure
T, p_Q	Threshold for samples network creation set by a chosen percentile p_Q
B^i	Sample-to-sample similarity network based on a distance metric which does not include species i
$b_{k,k'}^i$	Matrix elements of B^i; distance between samples k and k' not including species i
s_k^i	Labeling variable of sample k based on the presence/absence of species i
w^i	Total number of edges in the adjacency network B^i
d_k^i	The degree of node k in the adjacency network B^i
L^i	Longitudinal EPI of species i, based on the average distance of samples between two points of time.

Supplementary Table 1: Glossary of mathematical symbols and definitions used throughout the manuscript.

Supplementary Software

Directed Barabási-Albert networks

```matlab
function net = BA_directed(N, m_0, m, d)
% N - Total number of nodes
% n_0 - Number of seed nodes
% n - Maximum number of new connections (m≤m_0)
% d - Directionality parameter
% d=1: Undirected, d=0: Fully directed

% Initializing the network
net = false(N, N);
net(1:n_0, 1:n_0) = true; % Fully connected
net(1:N+1:end) = false; % Removing self-loops
net = digraph(net); % Creates MATLAB graph

% Adding N-n_0 nodes sequentially
for i = (n_0+1):N
    d_i = outdegree(net); % Degree of nodes
    d_total = sum(d_i);
    p_i = d_i./d_total; % Prob of edge to i
    chance = rand(1);
    if d > chance
        % Same in/out
        ind = randsample(N, n, true, p_i);
        net = addedge(net, i, ind);
        net = addedge(net, ind, i);
    else
        % Different in/out
        ind = randsample(N, 2*n, true, p_i);
        ind_out = ind(1:n);
        ind_in = ind(n+1:end);
        net = addedge(net, i, ind_out);
        net = addedge(net, ind_in, i);
    end
end
end
```

Empirical presence-abundance interrelation measures, D_1, D_2, Q

```
function D1 = EPI_D1(S)
%EPI_D1 Empirical Presence-Impact of a cohort by using the D1 measure
%   D1 = EPI(data) returns the D1 EPI values of each of the N species in a
%   cohort of M samples, using the Bray-Curtis dissimilarity measure.
%   S is a cohort of samples, represented by a N-by-M matrix of abundances.
%   D1 is an N-by-1 vector of D1 values.

% Initialization
[N, M] = size(S);
S_01 = double(S>0);
D1 = nan(N, 1);

for i = 1:N
    % If the species is always present/absent, D1 is undefined
    if sum(S_01(i, :), 2) ≠ 0 || sum(S_01(i, :), 2) ≠ M

        % Dividing into the two groups
        ind_pres = S_01(i, :) ≠ 0;
        S_pres = S(:, ind_pres);
        S_abs = S(:, not(ind_pres));

        % Removing the i species
        S_pres(i, :) = [];
        S_abs(i, :) = [];

        % Normalizing
        S_pres = S_pres./sum(S_pres);
        S_abs = S_abs./sum(S_abs);

        % Calculating D1
        D1(i) = sum(sum(pdist2(S_pres', S_abs', ...
            @BC_pdist2)))./(sum(ind_pres).*sum(not(ind_pres)));

    end
end

function Dpdist = BC_pdist2(ZI, ZJ)
    BC = @(x, y) sum((abs(x - y)))./(sum(x) + sum(y));
    m2 = size(ZJ, 1);
    Dpdist = nan(m2, 1);
    x = ZI;
    for k = 1:m2
        y = ZJ(k, :);
        Dpdist(k) = BC(x, y);
    end
end

end
```

```
function D2 = EPI_D2(S)
%EPI_D2 Emperical Presence-to-abundance Interrelation  of a cohort by using the D2 ...
    measure
%   D2 = EPI(data) returns the D2 EPI values of each of the N species in a
%   cohort of M samples, using the Bray-Curtis dissimilarity measure.
%   S is a cohort of samples, represented by a N-by-M matrix of abundances.
%   D2 is an N-by-1 vector of D2 values.

% Initialization
[N, M] = size(S);
S_01 = double(S>0);
D2 = nan(N, 1);

for i = 1:N
    % If the species is always present/absent, D2 is undefined
    if sum(S_01(i, :), 2) ≠ 0 || sum(S_01(i, :), 2) ≠ M

        % Dividing into the two groups
        ind_pres = S_01(i, :) ≠ 0;
        S_pres = S(:, ind_pres);
        S_abs = S(:, not(ind_pres));

        % Removing the i species
        S_pres(i, :) = [];
        S_abs(i, :) = [];

        % Normalizing
        S_pres = S_pres./sum(S_pres);
        S_abs = S_abs./sum(S_abs);

        % Calculating D2
        D2(i) = pdist2(mean(S_pres'), mean(S_abs'), @BC_pdist2);

    end
end

function Dpdist = BC_pdist2(ZI, ZJ)
    BC = @(x, y) sum((abs(x - y)))./(sum(x) + sum(y));
    m2 = size(ZJ, 1);
    Dpdist = nan(m2, 1);
    x = ZI;
    for k = 1:m2
        y = ZJ(k, :);
        Dpdist(k) = BC(x, y);
    end
end

end
```

```matlab
function Q = EPI_Q(S, threshold_net)
%EPI_Q Emperical Presence-to-abundance Interrelation of a cohort by using the ...
    modularity meausre, Q
%   Q = EPI(data) returns the Q EPI values of each of the N species in a
%   cohort of M samples, using the Bray-Curtis dissimilarity measure.
%   S is a cohort of samples, represented by a N-by-M matrix of abundances.
%   threshold_net is the distance threshold for the constructing of the
%   sample-to-sample network, by percentile
%   Q is an N-by-1 vector of Q values.

% Initialization
[N, M] = size(S);
S_01 = double(S>0);
Q = nan(N, 1);

for i = 1:N
    % If the species is always present/absent, Q is undefined
    if sum(S_01(i, :), 2) ≠ 0 || sum(S_01(i, :), 2) ≠ M

        % Removing the i species
        S_i = S;
        S_i(i, :) = [];

        % Normalizing
        S_i = S_i./sum(S_i);

        % Building the network
        distances_i = pdist(S_i', @BC_pdist2);
        [cdf_dist, dist] = ecdf(distances_i);
        dist_threshold = dist(find(cdf_dist≤threshold_net, 1, 'last'));
        B_i = squareform(distances_i, 'tomatrix')≤dist_threshold;
        B_i(1:M+1:end) = 0;
        s_i = double(S_01(i, :));
        s_i(s_i==0) = -1;
        s_i = s_i';

        % Calculating
        Q(i) = modularity(B_i, s_i);
    end
end

function Dpdist = BC_pdist2(ZI, ZJ)
    BC = @(x, y) sum((abs(x - y)))./(sum(x) + sum(y));
    m2 = size(ZJ, 1);
    Dpdist = nan(m2, 1);
    x = ZI;
    for k = 1:m2
        y = ZJ(k, :);
        Dpdist(k) = BC(x, y);
    end
end

function Qmod = modularity(B, s)
    B_graph = graph(B);
    d = degree(B_graph); % Degree of each sample
    q = B_graph.numedges;
    Qmod = (s' * (B - (d*d')/(2*q)) * s) / (4*q);
end

end
```

Longitudinal EPI, L

```
function [L, k_i] = LPI(S1, S2)
%LPI Longitudinal EPI of all the species using the samples in
%   the first and second collection (S1 and S2)
%   L = LPI(data) returns the L values of each of the N species in the two
%   cohorts of M subjects using the Bray-Curtis dissimilarity measure.
%   S1 is the cohort of samples in the first collection, and S2 is of the
%   second collection, represented by a N-by-M matrices of abundances.
%   L is an N-by-1 vector of L values. If the species had no reversed
%   presence-state in all the subjects, its L value is NaN.
%   [L, k_i] = LPI(S1, S2) also returns the number of subjects for which
%   the L value of each species was calculated for (the number of cases
%   where the species had a reversed presence-state).

[N, M] = size(S1);
L = nan(N, 1);
k_i = nan(N, 1); % Index number of samples
for i = 1:N
    L_i = [];
    k = 0;
    for j = 1:M
        data1 = S1(:, j);
        data2 = S2(:, j);
        datad1 = double(data1>0);
        datad2 = double(data2>0);
        % Checking if the presence-state of species i is reversed
        if datad1(i) ≠ datad2(i)
            % Removing the species
            data1_i = data1;
            data1_i(i) = [];
            data2_i = data2;
            data2_i(i) = [];

            % Normalizing
            data1_i = data1_i./sum(data1_i);
            data2_i = data2_i./sum(data2_i);

            % Calculating the distance before/after
            k = k + 1;
            L_i(k) = pdist2(data1_i', data2_i', @BC_pdist2);
        end
    end
    k_i(i) = k;
    L(i) = mean(L_i);
end

function Dpdist = BC_pdist2(ZI, ZJ)
    BC = @(x, y) sum((abs(x - y)))./(sum(x) + sum(y));
    m2 = size(ZJ, 1);
    Dpdist = nan(m2, 1);
    x = ZI;
    for kk = 1:m2
        y = ZJ(kk, :);
        Dpdist(kk) = BC(x, y);
    end
end

end
```
